# Computer Vision Applied to the Analysis of Pig Behavior Patterns in an Air-Conditioned Environment

**DOI:** 10.3390/ani16091353

**Published:** 2026-04-28

**Authors:** Maria de Fatima Araújo Alves, Héliton Pandorfi, Rodrigo Gabriel Ferreira Soares, Victor Wanderley Costa de Medeiros, Taíze Calvacante Santana, Vitoria Katarina Grobner, Gabriel Thales Barboza Marinho, Gledson Luiz Pontes de Almeida, Maria Beatriz Ferreira, Marcos Vinícius da Silva

**Affiliations:** 1 Department of Agricultural Engineering, Federal Rural University of Pernambuco, Dom Manoel de Medeiros Avenue, SN, Dois Irmãos, Recife 52171-900, Pernambuco, Brazil; fatima.alves@ufrpe.br (M.d.F.A.A.); gabriel.bmarinho@ufrpe.br (G.T.B.M.); gledson.almeida@ufrpe.br (G.L.P.d.A.); 2 Department of Statistics and Informatics, Federal Rural University of Pernambuco, Dom Manoel de Medeiros Avenue, SN, Dois Irmãos, Recife 52171-900, Pernambuco, Brazil; heliton.pandorfi@ufrpe.br (H.P.); rgfs@cin.ufpe.br (R.G.F.S.); victor.wanderley@ufrpe.br (V.W.C.d.M.); 3 Department of Agricultural Engineering, Federal University of Viçosa (UFV), Avenue Peter Henry Rolfs, Viçosa 36570-900, Minas Gerais, Brazil; taize.santana@ufv.br; 4 Chapadinha Science Center, Federal University of Maranhão, Chapadinha 65500-000, Maranhão, Brazil; mb.ferreira@ufma.br (M.B.F.); mv.silva@ufma.br (M.V.d.S.)

**Keywords:** animal welfare, automatic recognition, smart pig farming, technology in swine production

## Abstract

Observing pig behavior, such as feed intake, water intake, and resting behavior, is essential for improving the well-being of these animals. However, monitoring such behaviors by traditional methods can be exhausting for both humans and animals, interfering with their development. The research aimed to identify behavioral patterns of pigs in an air-conditioned environment through computer vision. Microcameras were installed in the animals’ stalls to generate videos over an experimental period of 92 days and the temperature and humidity of the air were simultaneously recorded. The physiological variables of the animals were collected to identify whether they were under heat stress. To recognize the drinking, eating, standing and lying behavior of pigs, YOLOv5 was trained and then the model was used to detect the animals. Regions in the images corresponding to the feeders and drinkers were established. To identify feeding behavior and water intake, criteria based on the occupation of the feeding zone by pigs detected in the standing position were established. The results showed that the trained model achieved an average accuracy rate of 97.3% and an average recall of 96.1% in animal detection. The model exhibited 97.5% accuracy and 97.0% recall rates in recognizing the feeding behavior and water consumption of pigs. The proposed method can be used in videos or images and minimizes the need for manual intervention, offering an efficient means of monitoring pig behavior in agricultural environments and contributing to the productivity of pig farming operations.

## Simple Summary

To ensure the welfare of pigs raised for food production, it is essential to observe their behaviors, such as feeding, drinking, and resting. However, human-based monitoring can be stressful for both the animals and the handlers themselves. This research developed an automated system using cameras and artificial intelligence to monitor pigs. The objective was to identify the animal’s behavioral patterns in a temperature-controlled environment without the need for direct human contact. The study lasted 92 days and used micro cameras installed in the pens. A computer program (YOLOv5) was trained to recognize, from images, when the pigs were drinking water, eating, standing, or lying down. Sensors also measured air temperature and humidity, and physiological data were collected from the animals to determine whether they were experiencing heat stress. The results were highly satisfactory: the system achieved over 97% accuracy in detecting the animals and recognizing feeding and drinking behaviors. This demonstrates that technology is highly effective. The main conclusion is that this tool can efficiently replace manual observation, reducing animal stress. For society, the impact is positive, as technology contributes to more ethical and productive food production, improving the welfare of animals in pig farming.

## 1. Introduction

Accurate and automated monitoring of pig behaviors, including feeding, drinking, and resting, is a key requirement for improving animal welfare in modern production systems, yet traditional manual observation methods are labor-intensive and may cause stress to the animals [1]. However, monitoring such behaviors by traditional methods can be exhausting for humans and animals, interfering with their development [2]. In addition, ref. [3] described that as production expands, it becomes unfeasible to observe all the activities of each animal, as well as to carry out its daily and individualized monitoring.

In addition, they may be flawed and may not accurately reflect the true health status of the animals [2]. Ref. [4] described that manual observation and recording of animal behavior involve significant effort and, in certain cases, may become unfeasible. Ref. [5] reported that conventional methods, such as painting, marking, ear tagging, and the application of radio frequency (RFID) systems, require substantial human and material resources, resulting in a considerable workload and reduced efficiency. According to [6], these methods are not suitable for healthy and accurate pig farming. 

Therefore, these methods need to be improved to achieve intensive and sustainable livestock development, as highlighted by [7]. To replace or enhance these methods [8] mentioned that producers must invest in accurate and effective solutions to the challenge of individual pig identification.

Thus, in order to monitor the behavior of pigs, many researchers have developed research with automated monitoring systems that employ sensors, such as infrared-sensitive cameras to monitor pigs’ activities in real time [9]. Nevertheless, ref. [10] reported that these types of sensor-based methods are susceptible to collision damage, which can lead to inaccurate identification and cause mental and physical stress to the animals. Other researchers have used camera-based surveillance systems to monitor the animals automatically [11,12].

Ref. [6] described that more recent deep learning techniques have been widely used for computer vision applications (object recognition, object classification and object detection), including methods based on deep neural networks.

Ref. [13] developed a model for recognizing feeding behaviors and water intake in pigs, using three different models: VGG19, Xception and MobileNetV2 and achieved a recall of over 97%. Ref. [14] presented an improved ResNAM network to recognize pig faces and achieved good results, with a 3% increase in recognition accuracy.

To recognize the feeding behavior and occupancy index of four sows, ref. [15] used a Faster Region-based Convolutional Neural Network (Faster R-CNN) and obtained good results.

However, these deep learning techniques require many parameters and require high computational costs to perform training and testing [6]. Ref. [16] mentioned that during training with convolutional networks using images, a very high Graphics Processing Unit (GPU) capacity is required. Ref. [17] stated that due to these computational requirements, deep learning-based methods have limited applicability on devices with reduced memory and processing capacity.

In parallel, ref. [18] mentioned that among computer vision techniques based on convolutional neural networks, the YOLO (You Only Look Once) tool for object detection has emerged as a faster and more efficient approach for training and can also be implemented in Google Colab using a free GPU. Furthermore, according to [19] YOLOv5 was developed with Python language, which can be another advantage. In addition, YOLOv5’s native framework is PyTorch v2.0.1, which allows for faster training [20].

According to [21], YOLO performs object detection in a single stage directly on the input image, thus presenting a faster execution time than two-stage object detection methods. According to [22], YOLOv5 adopts an approach in which a single convolutional network is employed to make simultaneous predictions, both bounding boxes (treating object detection as a regression problem) and of the probabilities associated with the classes of the detected objects (acting as a classifier).

The YOLOv5 architecture consists of three main parts: the backbone, neck, and head. The backbone (CSPDarknet53) extracts feature maps from the input image using cross-stage partial connections to reduce computational cost while preserving accuracy. The neck (PANet, Path Aggregation Network) aggregates features from different scales to improve the detection of objects of varying sizes. The head produces final predictions (bounding boxes and class probabilities).

Many studies have been developed in pig farming using the YOLOv5 algorithm. Ref. [23] used YOLOv5 to detect and count pigs in feeders and drinkers in real time. Ref. [24] used YOLOv5 to detect and count pigs in slaughterhouses. Ref. [25] used YOLOv5 to detect pigs under different lighting and occlusion conditions. Ref. [26] used the YOLOv5 to detect emotions in pig faces.

In recent years, advanced technologies such as sensing technology and the Internet of Things have experienced rapid development, being able to effectively acquire information about the physiological state, movement, behavior of animals, and information from the external environment, as reported by [27,28].

Thermal stress is a major welfare and productivity issue in pig farming, especially in tropical climates. Behavioral changes (e.g., increased lying, reduced feeding, increased drinking) are among the first indicators of heat stress, preceding measurable physiological changes. Therefore, integrating environmental monitoring with automated behavior recognition can provide early warning of thermal discomfort and guide management interventions.

Most of the studies using deep learning techniques have been studying the behavior of animals. However, the association between the thermal comfort of the environment and the behavioral ethogram is often not considered, even though both are influenced by the local microclimate. In this way, this work contributes to researchers and entrepreneurs by presenting a computer program capable of associating the production environment with animal behavior, without interfering with the animal’s normal activities, using images or videos from low-cost 2D monitoring cameras. 

Although YOLO-based behavior detection has been applied in livestock, most studies focus either on behavior classification alone or on thermal comfort separately, without integrating both dimensions. Furthermore, existing works rarely compare model performance across different housing environments (air-conditioned vs naturally ventilated) or directly link behavioral outputs to environmental and physiological indicators of heat stress. The main practical contributions of this study are: (i) the simultaneous use of YOLOv5 to recognize standing, lying, eating, and drinking behaviors in both air-conditioned and non-air-conditioned pens using low-cost 2D cameras; (ii) the quantitative association of behavioral time budgets with microclimate variables (temperature, humidity, BGHI) and physiological responses (rectal temperature, respiratory rate), demonstrating that automated behavior detection can reflect thermal stress; and (iii) the comparative evaluation of YOLOv5 variants (s, m, x) to identify the most suitable model for practical deployment in swine production, balancing accuracy and computational efficiency. While similar YOLO-based behavior detection systems have been reported, this study provides additional practical value by (a) validating the approach in two contrasting thermal environments, (b) integrating environmental and physiological data to interpret behavioral outputs, and (c) offering guidance on model selection for real-world farm settings. These contributions support the use of computer vision as a tool for heat stress monitoring, complementing rather than replacing existing welfare assessment methods.

## 2. Materials and Methods

### 2.1. Location of the Experiment

**Figure 1:**
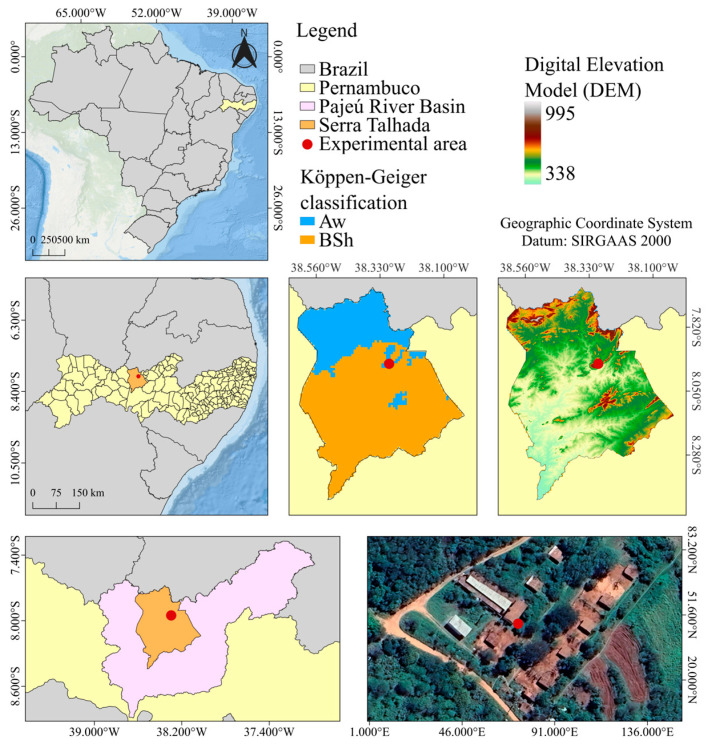
Geographic location of the experiment site.

The present study was conducted in the Vivarium for Experimentation with Swine, located on the campus of the Federal Rural University of Pernambuco (UFRPE), located in the city of Serra Talhada, in the hinterland region of the state of Pernambuco, Brazil. The geographic location of the municipality is delimited by the coordinate’s latitude 7°57’20.60″ S and longitude 38°17’45.22″ W, as illustrated in Figure 1.

According to the Köppen climate classification, the region is characterized by a BSwh-type climate (hot semi-arid, with dry winter and summer rainfall), which represents a hot and dry climate [27]. For reference, the classifications Aw (tropical savanna) and BSh (hot semi-arid) appear in Figure 1; these are neighboring climate zones that share similar temperature and rainfall patterns. The average annual temperature in Serra Talhada is 24.8 °C, and the average annual rainfall is 642.1 mm. The relative humidity of the air in the region is close to 62.5%. These climatic conditions result in an atmospheric demand of 1800 mm per year, with a deficit of 1143 mm per year [29].

### 2.2. Facilities

The research was conducted in an experimental shed with a total area of 330 m^2^. The shed was positioned in an east–west direction, which is a common practice in tropical regions to minimise the incidence of direct solar radiation inside the stalls during the hottest hours of the day, thereby reducing thermal load and improving environmental conditions. The stalls located inside the shed were built with masonry materials, with concrete floors and had dimensions of 2 × 3 m each. The roof had a unidirectional slope and was covered with ceramic tiles. In addition, the stalls were equipped with adequate feeders and drinkers to meet the needs of the pigs throughout the experiment.

### 2.3. Experiment

Data collection took place over 92 days, between August and December 2017. The experiment was conducted in accordance with ethical guidelines and was approved by the Ethics Committee on the Use of Animals of the Federal Rural University of Pernambuco (CEUA/UFRPE). The approval protocol used was n°. 23082.021090/2016-81, ensuring that all the necessary measures for the welfare and care of the animals were followed during the research.

A total of 18 pigs, including males and females, were distributed in groups of 3 in each stall. At the beginning of the experiment, the pigs had an average body weight of 30.4 ± 3.2 kg (mean ± SD), with no significant difference between treatments (*p* > 0.05). The animals were allocated to stalls in a completely randomised design, balanced for sex and initial weight across the six pens. Each stall contained a mix of males and females (approximately 2 males and 1 female per stall, or vice versa, depending on availability). The groups were homogeneous at the start, and no animal was removed or replaced during the 92-day trial. The animals were selected from a group available in the university’s experimental vivarium, from the commercial crossbreed 3/4 Duroc and 1/4 Pietrain in the growing phase. The pigs were fed twice a day, once in the morning and once in the afternoon and were allowed to drink at any time.

Six stalls were used in the study, three with an air conditioning system, equipped with air conditioners to study the effects of adiabatic evaporative cooling on the animals and another 3 pens without any air conditioning system (under natural ventilation), allowing the analysis of the animals under natural ventilation conditions. The air conditioners used in the stalls had an average flow of 3 L/h, with independent motors and propellers rotating at a speed of 1750 RPM and central disc at 3450 RPM.

### 2.4. Variable Collection

#### 2.4.1. Meteorological Variables

To characterize the microclimate of the internal environment of the facilities, air temperature (Tbs, in °C), relative humidity (RH, in %) and black globe temperature (Tg, in °C) were recorded during the 92 days of monitoring. The meteorological elements were recorded using a U12-012 (Onset Computer Corporation, Bourne, MA, USA) datalogger of the HOBO^®^ brand, during the day and night. Environmental data were logged every 15 min throughout the entire experimental period. The positioning of the instrument followed the recommendations of [30], which suggest that the equipment be installed preferably in the central region of the shed.

These variables were collected inside the stalls, using a datalogger and in the external environment, using a meteorological shelter. Each piece of equipment was positioned at a height of 1.50 m, to obtain representative information about the variations between the treatments.

#### 2.4.2. Physiological Variables

To evaluate the impact of different air conditioning systems on the thermal comfort of the animals, physiological variables, rectal temperature (RT), respiratory rate (RR) and skin surface temperature (TSP) were collected. These measures were carried out once a week, throughout the experimental period.

RT was obtained using a digital clinical thermometer inserted into the animals’ rectum. RR was measured by counting flank movements over 15 s, using a stopwatch, and then calculating the number of movements per minute. The TSP of the pigs was collected using a FLIR i60, Goleta, CA, USA, thermal imager. This equipment captured thermographic images of the animals’ entire bodies. The images were acquired at 1 m from the animal, following the recommendation of [31], who claim that this distance is enough to frame the entire body of the pig. In addition, the thermal imager settings were adjusted to an emissivity of 0.98, which, according to [32], is suitable for biological tissues and has been used in research with pigs. In addition, the values of temperature and relative humidity at the time the images were recorded were inserted as parameters for correcting the temperature of the thermal im-ages, allowing the analysis of the relationships between the body temperatures of the animals and the environment in which they were kept.

#### 2.4.3. Behavioral Variables

To monitor the behavior of the animals, 30-min videos were recorded at 25 frames s^−1^ (FPS) with an image frame width of 640 pixels and a frame height of 360 pixels. The recordings were made once a week, over a period of 24 h, throughout the experiment (92 days). The first week of analysis was excluded, because during this period the animals were adapting to the environments, as suggested by [33], resulting in 11 weeks, totaling 12 days of 24-h video recordings and 288 h of videos. 

To record the videos, microcameras (VMD S3020 IR, with a 90° viewing angle) equipped with 3.6 mm lens were used. These cameras were installed inside the stalls, at a height of 3.0 m from the floor, positioned approopriately to monitor the animals.

The analysis of the animals’ behaviors was carried out based on an ethogram adapted from [33,34,35]. Table 1 presents the classes of behaviors analyzed, the labels inserted for each class, and the observations regarding the behaviors evaluated.

This ethogram allowed the quantification of the frequency and percentage of time spent on each behavior listed, according to their respective treatments.

### 2.5. Methodological Procedure

#### 2.5.1. Assessment of Animal Thermal Comfort

The evaluation of the thermal environment was conducted through the analysis of the conditions of temperature and relative humidity of the air, observing the ranges necessary to meet the requirements of the animals [35,36], as shown in Table 2.

The evaluation of the thermal comfort of the animals was based on reference thresholds for rectal temperature (RT) and respiratory rate (RR) established in the literature for pigs under heat stress conditions [35] (Table 3).

The thermal characterization of the facilities was conducted using the Black Globe Temperature and Humidity Index (BGHI), following the methodology of [36], who developed the BGHI for finishing pigs (Table 4).

BGHI followed the methodology developed by [37] integrating black globe temperature and ambient humidity data, and is expressed by Equation (1):

BGHI = BGT + 0.36DPT + 41.5 (1)

where BGT is the black globe temperature (°C) and DPT is the dew point temperature (°C).

The values of temperatures (T) and humidity (RH) were used to estimate the temperature of the dew point by Equation (2).

(2) $$DPT=\frac{c*\gamma (T,RH)}{b-\gamma (T,RH)}$$

$$\gamma \left(T,RH\right)=es\left(T\right)*\frac{\left(RH\right)}{100}$$

where, DPT is the dew point temperature, γ (T, RH) is the ratio of the saturated vapor pressure to the current vapor pressure, b is a constant value of 238.88 and c constant value of 17.368, and es(T) is the saturated vapor pressure at a given temperature T.

#### 2.5.2. Monitoring Animal Behavior

**Figure 2:**
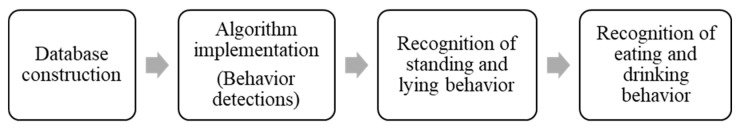
Flow chart of the Animal Monitoring Stage.

The monitoring of standing and lying down behaviors, eating and drinking was carried out in stages and is presented in the flowchart in Figure 2 below.

#### 2.5.3. Database Preparation

Eighteen videos of the experiment were selected, each lasting 30 min and having a rate of 25 FPS. Among these videos, 9 were from the stalls equipped with air conditioners, while the other 9 were from the stalls without air conditioners. These videos were submitted to the online platform Google Colab for the extraction of frames through codes.

After extracting the images, a manual observation was performed to identify those with significant behavioral differences. It was found that many images were practically identical, as they corresponded to sequential frames of the videos. Therefore, a manual selection of images that presented different behaviors was performed, following the procedure described by [38] and the recommendation highlighted by [20], who suggested that the diversity of the database is an essential feature and guarantees the generalizability of the YOLOv5 algorithm. From the 18 selected videos (each 30 min at 25 fps, for a total of 810,000 frames), we initially extracted one frame every 10 s, yielding 5400 frames. After removing near-identical frames (structural similarity index > 0.95), 5000 frames were retained. No images were discarded based on behavioral content; all behaviors present in the retained frames were annotated. The final dataset comprised 5000 images (15,000 instances), as described.

After the acquisition of the images, the preparation of the dataset was conducted in three stages: annotation of the data with their corresponding labels, pre-processing of the data, and application of the data augmentation technique to create synthetic images.

The annotation stage consisted of delimiting through bounding boxes the objects of interest in the images and pointing out the class to which each one belongs. The online platform Roboflow was used; this platform was also used in the work of [39] for annotation of images using convolutional neural networks. On the platform, the images were opened individually and, in each of them, rectangular boxes were manually drawn delimiting each pig within the image. Each box was labeled with a class indicating the animal’s behavior. 

The annotated labels were saved in YOLOv5 PyTorch format. In this format, each object in the image is described via a normalized bounding box location as follows (Equation (3)):

*class_id*, *x_axis_value*, *y_axis_value*, *width*, *heigh* (3)

where the term *class_id* corresponds to the class; *x_axis_value* and *y_axis_value* correspond to the *x* and *y* coordinates of the center of the bounding box; width and height correspond to the width and height of the bounding box [40]. 

This information was stored in an editable file with the corresponding name of each image and in a subsequent step the pixel coordinates of the eating and drinking classes were used. The annotations of all the images took about four weeks to complete. According to [11], annotating images of pigs in collective stalls takes, it takes about 5 min per image. Each animal was analyzed as an instance for the training of the algorithm.

As pre-processing steps, automatic orientation was applied to avoid errors in image orientation; the images were resized to 416 × 416 pixels to standardize their size according to the YOLOv5 input standard, and adaptive contrast equalization was applied [19]. For the purpose of training the model to recognize standing and lying behaviors only, the eating and drinking classes were temporarily merged into the standing category. The separate recognition of eating and drinking behaviors was performed later using the region intersection method described in Section 2.5.5, rather than by direct classification of those classes by YOLOv5.

As data augmentation techniques, image mirroring was applied, in which the images were flipped horizontally in the left/right direction and vertically in the up/down direction, as well as rotation of the images by 90° clockwise, counterclockwise and upside down. Data augmentation (horizontal/vertical flips, 90° rotations) was employed to increase dataset diversity and reduce overfitting, following standard practices in deep learning for animal detection [39,40].

Subsequently, the images were divided into distinct sets: 80% (4000 samples) were allocated to the training set, 10% (500 images) made up the validation set, and the remaining 10% (500 samples) were allocated to the test set. This procedure totaled 5000 images and 15,000 instances used in the study. The split into training (80%), validation (10%), and test (10%) sets was performed at the video level, not the frame level, to ensure that frames from the same video did not appear in both training and test sets. This guarantees independence and avoids data leakage.

This information is in accordance with the guidelines of [20], which recommend using diverse data sets, including different lighting conditions such as morning, afternoon, and night, and data from different cameras. The images obtained were analyzed and classified into specific behaviors, based on previous studies conducted by [33,34,35].

#### 2.5.4. Algorithm Implementation

The training was carried out in the Google Colab environment, using the open-source notebook made available by the YOLOv5 developer at the link https://github.com/ultralytics/yolov5 (accessed on 17 September 2024). The training was performed using Google Colab, a cloud-based platform that provides free access to GPU resources (Tesla T4, 15 GB RAM). This environment allowed efficient parallel processing via Compute Unified Device Architecture (CUDA).

Initially, a folder was created in the drive associated with the email to accommodate the files resulting from the training. Then, the project was set up in the Google Colab environment through access to the search platform. 

To start the training, the system was connected to a GPU and enabled the CUDA API was enabled, which allowed parallel processing and contributed to a more efficient and faster execution of the model training. 

Next, YOLOv5 was cloned directly from the developer’s GitHub repository, along with all its dependencies and necessary requirements. After the implementation of YOLOv5, the dataset was downloaded directly from Roboflow to the collaborative environment (Colab). Then, the.yaml file was configured to contemplate the four classes, since the original YOLO architecture is standardized for the classification of 80 categories. The training of the YOLOv5 model was conducted using the PyTorch v2.0.1 framework. 

Among the four available model variants, YOLOv5s (small model), YOLOv5m (medium model), YOLOv5l (large model), and YOLOv5x (extra-large model), it was decided to evaluate the small, medium, and large model to reduce computational cost.

YOLOv5 was chosen because it offers a favourable trade-off between detection accuracy and inference speed, and it has been successfully applied in numerous livestock monitoring studies [22,23,24,25]. Moreover, its native implementation in PyTorch v2.0.1 and compatibility with free GPU resources (Google Colab) make it accessible for research settings with limited computational budgets. Prior to the main experiment, we conducted a preliminary comparative test using a subset of 500 annotated images to evaluate YOLOv5 against two other single-stage detectors (YOLOv4-tiny and EfficientDet-D0). YOLOv5s achieved a mAP50 of 94.2% on this subset, compared to 89.6% for YOLOv4-tiny and 91.3% for EfficientDet-D0, while maintaining the fastest inference time. Based on these preliminary results and the existing literature, we selected YOLOv5 as the primary model.

Before starting the training, the hyperparameters were optimized using the method of genetic algorithms. This process, as suggested by [20] involved the creation of a base scenario, from which the hyperparameters were optimized. The base scenario was submitted to 10 training periods, in accordance with the recommendation of [20]. For the evolution of the hyperparameters, mutation with a probability of 80% and a variance of 0.04 was used, generating new offspring through the combination of the best parents of all previous generations. The offspring with the highest fitness were preserved at each generation of the evolutionary process and used to train the algorithm. The hyperparameters obtained were a initial learning rate of 0.0001, a momentum rate of 0.97 and a learning decay rate of 0.95. Other hyperparameters were also adjusted, including a batch size of 16 images, and the number of iterations set to 100 epochs. The model training was performed sequentially, following the steps available in the adopted notebook, using the hyperparameters obtained in the previous step.

#### Evaluating Model Performance

The experimental configuration was tested on an Ubuntu 22.04.3 LTS (Long-Term Support) system equipped with a six-core, 12-thread AMD Ryzen 5 2600 processor and an NVIDIA GeForce RTX 2060 8 GB graphics card. On the local system, training required 5 h for 100 epochs. Due to the computational demands of these procedures, Google Colaboratory (Colab) was also used. In Google Colab, the experiments in this study were conducted using Python 3.10.12, PyTorch v2.0.1 + cu118, and CUDA:0 (Tesla T4, 15,102 MiB), making use of the available free GPU, where processing for 100 epochs also required 5 h.

This GPU provided a significant increase in computational capacity, reducing training time and accelerating the training process. Similar studies have explored the use of GPUs to optimize training time and improve performance in computer vision tasks using machine learning, such as [41], which, in their behavioral analysis of pigs, also relied on machines equipped with GPUs. Ref. [12] used a computer with an 8 GB NVIDIA GeForce GTX 2070 GPU to recognize feeding and water consumption behaviors in pigs. Similarly, ref. [42] used GPUs in the analysis of swine activities. 

After applying the genetic algorithm technique over a total of 15 generations, which required 14.58 h using Colab’s NVIDIA A100-SXM4-40GB, the resulting values were implemented in the YOLOv5 model training process. The results obtained from these iterations yielded the following hyperparameters: an initial learning rate of 0.0082, a momentum coefficient of 0.9540, and a weight decay coefficient of 0.0005. The algorithm was trained using the Adam optimizer with a batch size of 16 images. Ref. [43] also explored the application of the Adam optimizer in their study on bounding box detection in pigs to investigate detection speed and accuracy. Similarly, ref. [44] used the Adam optimizer to perform detections using the YOLOv5 model in laying hens, conducting a 100-epoch training. All experiments in this study employed the same hyperparameters mentioned above. ReLU and Softmax were used as activation functions.

Table 5 provides information on the training duration required to recognize animal behaviors for each of the models analyzed, as well as the training time with and without GPU usage.

The performance of the model was evaluated using metrics commonly employed in deep learning, following the approach adopted by [45]. This includes metrics such as accuracy or precision (P), recall (R), average accuracy (AP), and mAP (mean average precision across all classes). *TP* (true positives) is the number of pigs correctly identified, while *FP* (false positives) is the number of pigs incorrectly identified and *FN* (false negatives) is the number of individuals that the model could not identify. *AP* represents the average precision. The equations of these metrics are expressed mathematically in Equations (4)–(6).

(4) $$Precision\;(P)=\frac{TP}{TP+FP}$$

(5) $$Recall\;(R)=\frac{TP}{TP+FN}$$

(6) $$mAP=\frac{1}{n}\sum_{i=1}^{n}APi$$

To evaluate the performance of the proposed method for drinking and eating behavior, videos recorded after feeding time were selected. A video of each stall was selected in the morning and another in the afternoon. For drinking behavior, the videos in which this activity was observed were divided into several segments.

Three metrics were used, as indicated by [46,47,48]. These metrics include the Mean Absolute Error (MAE) which represents the measure of the absolute difference between the predicted and actual variables, the Root Mean Squared Error (RMSE), which measures the dispersion of errors relative to the mean, and the coefficient of determination (R^2^), which reflects the quality of the models’ fit to the data, such that, the closer to 1, the more accurate the model. The analysis of these metrics considered the true boxes and the results of the intersections between the model detections and the regions of interest. Mathematically, these metrics are expressed by Equations (7)–(9).

(7) $$\mathrm{MAE}=\frac{1}{n}*\sum_{1-n}^{n}\left|\gamma_{True}-{Ŷ}_{pred}\right|$$

(8) $$\mathrm{RMSE}=\sqrt{\frac{1}{n}*\sum_{1-n}^{n}{\left(\gamma_{True}-{Ŷ}_{pred}\right)}^{2}}$$

(9) $$R^{2}=1-\frac{\sum_{1-n}^{n}(\gamma_{True}-{Ŷ}_{pred}{)}^{2}}{\sum_{1-n}^{n}(\gamma_{True}-\bar{y}{)}^{2}}$$

where *n* = number of samples in the test set, $\gamma_{True}$ is the actual value, ${Ŷ}_{pred}$ is the predicted value e and $\bar{y}$ is the average of the observations.

To verify whether there was a significant difference in the performance of the different versions of the YOLO model (YOLOv5s, YOLOv5m, and YOLOv5x), a one-way analysis of variance (ANOVA) was performed. The dependent variables were precision, recall, F1 score, and accuracy (mAP50) obtained from the test set for each model. For each model version, five independent training runs were conducted (i.e., five replicates per model) using the same training/validation/test split but with different random initializations. The assumptions of normality (Shapiro–Wilk test) and homogeneity of variances (Levene’s test) were checked and met (*p* > 0.05). The ANOVA was followed by Tukey’s HSD post hoc test when the overall F-test was significant. The significance level was set at α = 0.05.

#### 2.5.5. Recognition of Standing and Lying Behavior

**Figure 3:**
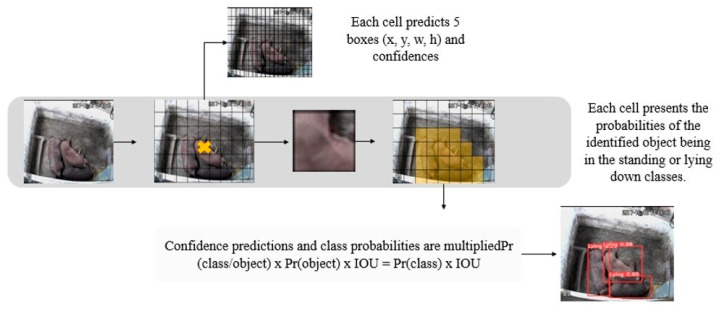
The process of detecting the behaviors of lying down and standing.

The behavior of standing and lying down was identified using the trained model, where an image was submitted for inference, as shown in Figure 3.

From this image, the model’s neural network divided it into 19 × 19 regions, as shown in step 1 in Figure 3. Each region was responsible for predicting 5 boxes, with the location of the animals (x, y, w, h) and the confidences of each box containing an animal displaying the behavior of standing or lying down (step 2 of Figure 3). In this way, this process provided a probability value for each of the possible classes that the algorithm was trained to find. Most of the boxes detected had a very low degree of confidence. 

To eliminate most of the boxes, a confidence threshold of 70% was assigned, so that all the boxes that presented lower values were removed and only those with higher values remained, according to step 3 (Figure 3). Finally, the boxes that remained on top of the image were responsible for the detection.

#### 2.5.6. Recognition of Feed and Water Intake

To recognize the eating and drinking behavior of pigs, the same criterion used in the study conducted by [49] was adopted, in which a pig was considered to be feeding when its head is in-side a feeder.

**Figure 4:**
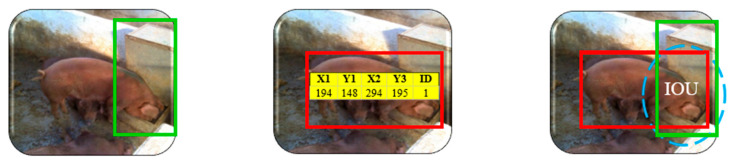
Recognition of eating and drinking behavior.

As pigs eat and drink in feeders and drinkers, these regions were delimited in each image using the OpenCV library. In this way, a correlation of the animals with these regions of interest was made. Feeding behavior is correlated with the feeder and drinking behavior with the drinker. In view of the situation in which the feeder may be empty and the animals explore this region; the evaluation of feeding behavior was analyzed only at the time of feed distribution. The recognition of eating and drinking behavior is shown in Figure 4.

**Figure 5:**
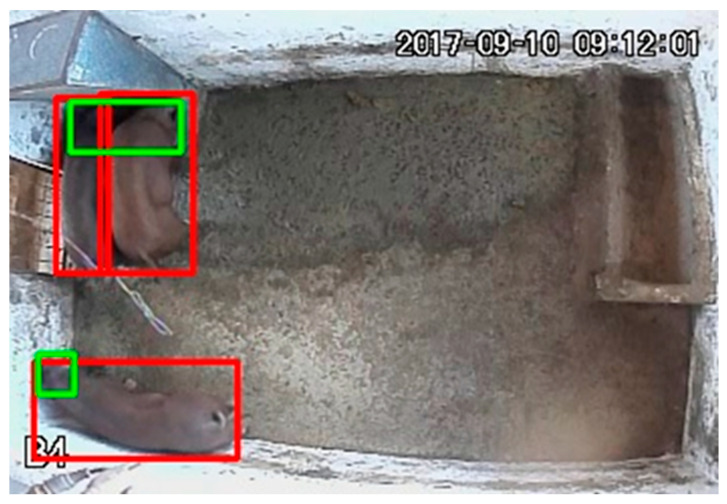
Recognition of eating and drinking behavior.

The regions of the feeder and drinker were delimited. Subsequently, through the detections made by the YOLOv5 model in the previous step, the coordinates of the bounding boxes associated with the standing class were captured, since the pigs stand while eating or drinking. In a subsequent stage, these coordinates were incorporated into a code developed in Python language, intended to analyze the feeding behavior of the animals. When a bounding box labeled as standing intersects with the feeders, it is possible that feeding behavior occurs, when it intersects with the drinkers, it is possible that water ingestion occurs. That is, the image is inserted for the model to identify the animals. The model detects the animals and delimits them with the bounding boxes (red color) (Figure 5). Feeding behavior occurs when the bounding boxes cross the regions of interest (green color) (Figure 5), combined with the feeding time; thus, the feeding behavior is recognized. When the bounding boxes intersect the drinking area, there is water intake, as shown in Figure 5.

As there are situations in which the animals approach the regions of interest and do not ingest food and water, a measure of the occupation of the zone by the detected pigs was suggested, indicating the percentage of the total area destined to food or drink that is effectively being used by this specific part of the animals. In this methodological procedure, the Occupation of the Areas of Interest (OC) was determined by the delimiting rectangles detected by the model. The calculation of the proportion of the area of the feeding zone that is being occupied by the pig bounding boxes was performed using Equation (10) as a reference.

(10) $$\mathrm{OC}=\frac{Model\;detections\;\Omega \;region\;of\;interest}{Region\;of\;interest}$$

where the numerator refers to the region where the pigs and the feeding zone overlap, or the specific part of the feeding zone being occupied by the pig bounding boxes, while the denominator represents the total area destined to pig feed.

Segments of 5 min were extracted from the 30-min videos after the delivery of feed and the number of frames with feed intake was acquired; then, the time of occupation at the feeder was obtained. To evaluate the performance of the recognition of eating and drinking behavior, accuracy and recall were calculated.

Thus, the monitoring of the animal behaviors was carried out considering the automatic outputs of YOLOv5, which automatically provided the information on pig detections, including the coordinates of the bounding boxes, the number of animals detected, the behavior class, the processing time of each frame (*tp*), the inference time (*ti*), and the non-maximum suppression time (*tsnm*) performed in each frame. In this way, it was possible to count the animals, the activities in each frame of the video, and the time spent on each activity automatically.

To quantify the time of the standing and lying down activities, the total number of frames and the sum of the information provided by the model were used, including the processing time (*tp*), the inference time (*ti*), and the non-maximum suppression time (*tnms*) for each frame according to Equation (11), to obtain the frame per second (FPS) rate achieved by the model. Then, the time was calculated for each of the classes observed by Equation (12).

(11) $$\mathrm{FPS}=\frac{1000}{\left(tp+ti+tnms\right)}$$

(12) $$\mathrm{Time}\;(H)=\frac{FPS}{\left(3600\right)}$$

The results were compared with the visual observation time for each of the activities of standing (walking and investigating) and lying down (sleeping and lying down), during the entire period analyzed. 

For eating and drinking behavior, the feeding and water intake time was accounted for by the Intersection over Union (IOU) of the animals’ bounding boxes with those of the feeders and drinkers. To quantify the duration of these intersections, the time was recorded in number of frames in which the areas of the feeders and drinkers were occupied by the bounding boxes of the animals in at least 50% of the rectangle corresponding to the activities in the standing position.

The manual analysis process was accompanied by videos and the activities performed by the animals while standing were manually recorded, including the activities developed in this posture, such as drinking, eating, walking and investigating. The behavior of lying down included the animals lying down and sleeping. The time that each animal spent on each activity was recorded. Subsequently, the data were tabulated in Microsoft Excel 2016 and graphs were made.

## 3. Results and Discussion

The results are organized as follows. First, we characterize the environmental conditions (temperature, humidity, BGHI) and the physiological responses (rectal temperature, respiratory rate) of pigs in the two housing treatments (Section 3.1 and Section 3.2). These data establish that the air-conditioned pens provided thermoneutral conditions, while the naturally ventilated pens induced heat stress. Next, we evaluate the performance of the YOLOv5 model in detecting standing, lying, eating, and drinking behaviors (Section 3.3 and Section 3.4). Finally, we integrate these behavioral outputs with the environmental and physiological data to demonstrate how the model can detect climate-induced behavioral changes (e.g., reduced feeding, increased drinking under heat stress), thereby validating its utility for heat stress monitoring.

### 3.1. Analysis of the Animals’ Environment

The results of the descriptive statistical parameters of the environmental variables collected for each scenario analyzed during the experimental period are presented in Table 6.

In the facilities, it was observed that the temperature followed a cyclical pattern throughout the day, reaching its maximum value during the afternoon and minimum in the morning. On the other hand, the relative humidity of the air showed an inversely proportional variation, registering its minimum when the temperature reaches its peak and vice versa. These patterns are documented in Table 5. This observation was also corroborated by the research of [50], who, when studying the thermal comfort of calves in a tropical climate, observed this same pattern of higher temperatures with lower relative humidity during the afternoon and lower temperatures in the morning, with higher humidity.

The meteorological records collected from the animals’ facilities during the experimental period showed, in the air-conditioned environment, a minimum temperature of 21.02 °C, a maximum temperature of 24.87 °C, a minimum relative humidity of 70.30%, and a maximum relative humidity of 85.73%. 

The average temperature observed in the environment with air conditioning was 22.59 °C. According to [51], the ideal air temperature for pigs in the growing and finishing phases is between 18 to 21 °C. According [52], the air temperature considered comfortable is between 18 and 25 °C. In the present study, the zootechnical facilities equipped with air conditioners provided comfortable conditions for the animals, the analyzed variables of dry bulb temperature did not exceed the limit range as proposed, for example. Ref. [51] and [52], framing the environment within the zone considered thermoneutral for animals in the growth and finishing phase. The results obtained in this work are close to those found in the research conducted by [33], which found temperature values between 18 and 26 °C for pigs growing in air-conditioned environments.

The average relative humidity for the environment with air conditioners was 81.99%. The average relative humidity in this study exceeded the ideal range between 50 and 70% considered by [51] for pigs in this phase of growth and finishing. Ref. [53] They also consider the relative humidity of the air to be optimal for pigs in the range of 50 to 70%. 

When pigs are exposed to a thermoneutral environment, according to [54], they do not require additional energy expenditure to regulate their body temperature and their productive functions occur optimally, free of stress and thus the animals have better productive performances. 

In the stalls with natural ventilation, the minimum temperature recorded was 24.56 °C and the minimum relative humidity was 46.62%. The maximum temperature was 33.52 °C and the maximum relative humidity was 74.63%.

The average air temperature in these facilities was 30.46 °C, exceeding the proposed limit range by [51,52]. These facilities did not provide comfortable conditions for the animals. The dry bulb temperature was higher than the thermoneutral zone of the animal; therefore, the pigs were under thermal stress conditions during the experimental period. 

The average relative humidity for these facilities was 54.62%, considered within the desired values of 50 to 70%. However, the higher temperatures made this the most stressful environment for the animals.

### 3.2. Analysis of Physiological Variables

The results of rectal temperature (RT) and respiratory rate (RR) of the animals in stalls with and without air conditioners are presented in Table 7 below.

According to Table 7, the animals in the stalls with air conditioners showed mean rectal temperature (RT) ranging from 37.90 °C to 38.40 °C and an average respiratory rate (RR) of 44.67 to 59.67 mov·min^−1^. In the research of [55] the average RT found for pigs evaluated in a thermal environment within the thermoneutral zone (18 to 25 °C) was between 36.19 °C and 38.8 °C [56]. According to [57], The rectal temperature when pigs are in the thermoneutral zone is on average, 38.85 to 39.42 °C. Ref. [58] considers that pigs are in thermal comfort when they have RT ≤ 39.3 °C and RR ≤ 60 mov·min^−1^ and above these thresholds, they are in discomfort. Thus, the results indicate that the animals in these facilities were, in fact, in thermal comfort.

As shown in Table 6, the animals housed in the stalls without air conditioners had rectal temperature (RT) values ranging from 38.50 °C to 38.75 °C, while the respiratory rate (RR) ranged from 48.33 to 73.50 mov·min^−1^. In the research of [56] pigs subjected to heat stress showed RT ranging from 39.39 to 39.46 °C and RR ranging from 82.21 to 83.23 mov·min^−1^. Ref. [59], investigating physiological variables of pigs in hot environments, they found RR ranging from 56.6 to 71.5 mov·min^−1^. Ref. [60] identified respiratory rate values ranging from 6.2 to 107.3 mov·min^−1^ in pigs under heat stress [61] found RR values of 88, 89 and 73 mov·min^−1^ in pigs housed in hot climates. The rectal temperature of the animals remained within the indicative range of comfort; however, the respiratory rate and environmental variables revealed a situation of thermal discomfort. This occurred because in situations of heat stress, the respiratory rate of pigs tends to increase before the rectal temperature reaches levels considered uncomfortable, as observed by [62].

The animals presented higher RT values than those of the stalls with air conditioners and RF values higher than 60 mov·min^−1^ respectively, which exceeded the values considered ideal for these physiological variables and proved that the animals were under conditions of heat stress during the study for these facilities. This is because the respiratory rate of pigs increases in environments above the thermoneutral zone according [59].

Physiological variables, such as respiratory rate and rectal temperature, exhibited a pattern similar to that observed in the study conducted by [63], presenting lower values in the morning and maximum values during the midday and afternoon. Ref. [41] pointed out out that as the air temperature increases, the respiratory rate also increases.

Regarding the Black Globe Humidity Index (BGHI), used to assess heat stress in animal production environments, this study adopted the criteria established by [36]. According to these criteria, the environment is under thermal comfort when the ITGU is between 68 and 74, enters the alert range between 74 and 76, and reaches the emergency phase when it exceeds 76, specifically for pigs. The results of this analysis are presented in Table 8.

In the environment with air conditioning, the data analysis revealed that the mean BGHI was 71.45, with a minimum value of 69.12 and a maximum value of 74.8, classifying the environment as comfortable and alert in terms of thermal comfort level. It was observed that the environment was classified as being in the alert phase from 1:40 p.m. to 1:50 p.m. This may be related to the maximum temperature recorded at these times, which reached 24.51 and 24.87 °C, thereby requiring specific interventions to ensure the comfort of the animals. The other time periods remained at optimal levels for the well-being of the pigs. Thus, it was observed that the animals were in the comfort phase during 95% of the evaluation period (from 8:00 a.m. to 4:00 p.m.), since only 5% of the time (from 1:30 p.m. to 1:40 p.m.) the environment was classified within the alert range. Therefore, the animals spent most of their time in a condition of thermal comfort. 

In the environment without air conditioning, the BGHI values were recorded at an average of 79.82, with a minimum value of 73.63 and a maximum value of 83.17. These results show that, on average, the thermal comfort conditions were above the ideal conditions for the well-being of the animals. The minimum value was found in the first record, when the temperature was still low, but with the increase in temperature the BGHI was classified as being within the alert range and later, at 8:40 am, the index indicated an emergency condition. Thus, the animals were determined to be in emergency conditions most of the time. This suggests that the stalls without air conditioners could not minimize the effects of the local temperature, given the climatological conditions of the region, and that these environmental conditions were not favorable for the thermal comfort of the pigs, indicating that measures were necessary to improve the environment and ensure the well-being of the animals.

The clear differences in BGHI, respiratory rate, and rectal temperature between the two housing treatments confirm that the non-air-conditioned pens imposed significant heat stress. These physiological and environmental differences provide a reference for interpreting the behavioral outputs of the YOLOv5 model in the following sections.

### 3.3. Monitoring Animal Behavior

#### 3.3.1. Evaluation Model Performance

In the work of [19], YOLOv5x was also the version that required the longest training time, even with a smaller number of images, but presented the best results.

**Figure 6:**
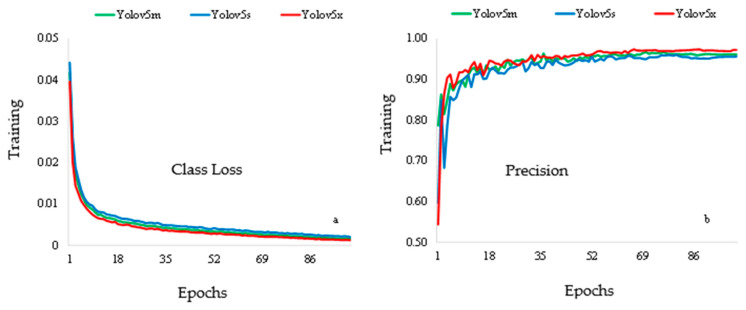
Training Curve: (**a**) Class Loss Curve and (**b**) Accuracy.

The results obtained during the training of YOLOv5 for the behavior classes analyzed by the different versions of YOLOv5 can be observed by analyzing the evolution of the loss function for the classes (Figure 6a) and the precision (Figure 6b) throughout the training epochs.

The training process of YOLOv5s began with a class loss (cls_loss) of 0.04 for the YOLOv5m and YOLOv5s versions, and 0.03 for the YOLOv5x (Figure 6a). As training progressed, at epoch 98, the loss was significantly reduced to 0.001 for YOLOv5s, and to 0.002 for YOLOv5m and YOLOv5x, and then stabilized. Similar results were found in the research conducted by [42], when the epoch was in the range of 75 to 100, the loss associated with the different versions of YOLOv5 decreased and stabilized at about 0.015 to 0.03. This indicates that initially the model was learning to map the input data to the correct classes. As the training progressed, the loss diminished as the model adjusted its parameters to fit the data. This reduction in loss shows that the model converged, as the error began to stabilize at values close to 0.001 and did not decrease further after subsequent training epochs, without underfitting or overfitting. 

In Figure 6b, it can be observed that the behavior of the precision in the training curve improved when the model converged (at epoch 98), with accuracy of 96.11% and 95.58% for YOLOv5m and YOLOv5s, respectively, whereasYOLOv5x obtained 97.21% at epoch 97 and remained at this values until the end of training. Similar patterns of evolution in the training curve were identified in the study conducted by [24] who noticed an improvement in the detection accuracy of the model before reaching 100 epochs. This indicates that all versions of YOLOv5 performed well in training and were able to converge on a solution that minimizes loss. In addition, this shows that the models were well adjusted to the training data and were able to make more reliable predictions about the behavior classes of the animals present in the images.

As seen in Figure 6b, the accuracy of the model improves as the training epochs increases. This behavior of precision was also observed in earlier research, when YOLOv5 was used to recognize the eating, standing and lying classes in cattle.

The results obtained were similar to the findings of [43]. In this study, when the training reached convergence, the average accuracy was stable at approximately 97%, revealing consistency in the results obtained. This similarity to the results reported by [43] indicates that the models proposed in this work have the potential to achieve a promising performance in object detection and computer vision, corroborating the conclusions of the reference study.

**Figure 7:**
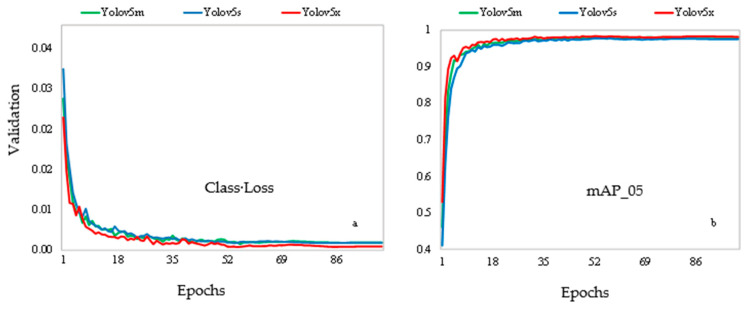
Validation curve: (**a**) class loss curve and (**b**) mAP_05 in validation.

The validation class loss was used to evaluate the accuracy in detecting pig behaviors in the images. Figure 6a illustrates the class losses and Figure 7b the mAP_05 in the validation set after each epoch during the validation process.

Figure 7a shows a decreasing pattern throughout the training of the models, indicating continuous progress in performance as the models learned, but with fluctuations. These fluctuations, according to the study of [24], occurred because there are significant differences in the behavior of the animals. The effect of detection performance is reflected by the increase in mAP50% in Figure 7b. Correspondingly, by the time the models converged, the YOLOv5 models obtained a mAP of 96.71 and 98.11% and stabilized.

Evaluating Figure 7, it can also be seen that during the process of training the detection model, as the number of epochs increased, a progressive decrease in the slope of the class loss curve in the validation set was observed, culminating in stabilization. As the epochs progressed, variations were observed in the results of the localization loss, confidence loss, and classification loss metrics. The results obtained demonstrate that the accuracy and recall metrics reached stability from epoch 98, with values of 97% and 96%, respectively. In addition, the mAP values for the 0.5 threshold were 98% as illustrated. This stabilization of the metrics indicates that the model achieved consistent performance in detecting objects after this point in training.

It is common to evaluate the performance of computational models in the test set. In the research of [44] approximately 800 images were used in the test set to evaluate the results of the YOLOv5 performance. In the present work, a total of 500 images were used in the test set. The results obtained in the test set are presented in Table 9.

As presented in Table 9, the results demonstrate that the YOLOv5m model achieved an accuracy of 0.982, indicating that it was able to correctly identify 98.2% of the instances of this class, with a recall of 98.0%. In addition, the mAP50 metric, which represents the average accuracy with a 50% confidence threshold achieved 99.2%, while the mAP50-95, which evaluates the average accuracy over a wider confidence range (50 to 95%), obtained a value of 94.8%. For standing behavior, the model recorded an accuracy of 94.8% and recall of 94.2%. The mAP50 and mAP50-95 metrics reached 97.7% and 78.4%, respectively, and the average values were accuracy of 0.965, recall of 0.961, mAP50 of 0.984, and mAP50-95 of 0.866.

Similar results were identified in the study by [5], who also conducted experiments with YOLOv5m for the detection of pig heads, achieving an accuracy of 96.10%, a recall of 86.70%, and mAP05 values of 93.50% and mAP95 of 94.20%, demonstrating the potential of the algorithm in applications in animal production.

In the research conducted by [64], the researchers found an accuracy of 99.3% and 99.1%, with a recall of 98.1% for the detection of laying hens on commercial farms using YOLOv5. In the study of [23], the authors found an accuracy of 0.989, a recall of 0.996, mAP0.50 of 0.994, and mAP0.50:0.95 of 0.796 to detect pigs using YOLOv5m.

The results highlight the effectiveness of the model in analyzing and identifying patterns in a representative set. These metrics serve as fundamental indicators for evaluating the overall performance of YOLOv5. In Table 10, the accuracy, recall, mAP50, and mAP50-95 of the YOLOv5s model for the test set are displayed.

For the lying behavior, the model demonstrated an accuracy of 0.984 (98.4%), indicating the precision of the detections related to this behavior, and recorded a recall of 0.981 (98.1%), which means that it identified 98.1% of the cases of this behavior. The mAP50 metric reached 0.985 (98.5%), while the mAP50-95 obtained a value of 0.975 (97.5%). In the case of standing behavior, the model showed an accuracy of 0.962 (96.2%), with a recall of 0.941 (94.1%). The mAP50 and mAP50-95 metrics reached 0.985 (98.5%) and 0.952 (95.2%), respectively. The mean values for the animals’ behaviors were accuracy of 0.973, recall of 0.961, and mAP50 and mAP50-95 of 0.985 and 0.963. This shows that by increasing the confidence threshold, the mAP50-95% decreased. 

In a study conducted by [24] to detect pigs, the authors achieved an mAP50 of 95.2% and an mAP50-95 of 65.83%. The reported mAP50 of 95.2% indicates a high level of accuracy in detecting swine-related objects using the YOLOv5 model, with a confidence threshold of 50%. However, the decrease of mAP50-95 to 65.83% suggests that as the confidence threshold is extended to a wider range (50 to 95%), the model’s performance in terms of average accuracy decreases, indicating that within a wider confidence range of 50 to 95%, the mAP values for YOLOv5s were lower. This behavior of mAP50-95 is consistent with the results obtained in the present study, providing additional support for the efficacy of the YOLOv5 model in studies with pigs. 

Table 11 presents the YOLOv5x evaluation metrics, obtained from the analysis of the test dataset for the different classes of animal behavior.

For the lying class, a precision (P) of 0.987 was observed, indicating that the model was able to correctly identify 98.7% of the instances related to this class. The recall associated with this class was 0.984 (98.4%), which demonstrates a satisfactory recall rate. The mAP50 and mAP50-95 metrics were calculated at 0.993 (99.3%) and 0.969 (96.9%), respectively, reinforcing the satisfactory performance of the model for this class. The standing class revealed an accuracy of 0.962, highlighting that the model was correct 96.2% of the time when the animals were performing activities while standing. The recall, calculated at 0.941 (94.1%), suggests a good ability to recover from positive examples. The mAP50 and mAP50-95 metrics, with values of 0.969 (96.9%) and 0.744 (74.4%), indicate the consistent ability of the model to identify the behaviors of the standing animal. The mean values for the model were accuracy of 0.974, recall of 0.962, mAP50 and mAP50-95 of 0.981, and 0.852. In the research of [65], the accuracy was 0.99 for detecting the behavior of standing pigs, whereas lying pigs the accuracy was 0.98 and the mAP was 0.95. In the research of [23] to perform pig detection and counting, the accuracy of yolov5x was 0.989, recall of 0.996, mAP0.50 of 0.994 and mAP0.50:0.95 of 0.796. In the research of [66], the YOLOv5 method demonstrated high accuracy in the recognition of standing, prone and lateral lying behaviors, with values in the validation set reaching 99.1, 95.3 and 97.4%, respectively.

Overall, as confidence levels were raised, forecasts went through a gradual filtering process, resulting in reduced mAP_05:95 metrics. This indicates that by increasing the level of confidence required to consider a detection as valid, the model has become more conservative in its predictions, resulting in fewer accepted detections, but with greater confidence in their accuracy. This result was also observed by [67]. This procedure is useful for controlling the number of false positive detections and for improving the overall quality of predictions in scenarios with high reliability requirements. However, it’s important to note that by imposing a stricter threshold, the model may also miss some true detections, which may explain the reduction in metrics, accuracy, and recall. 

This result highlights the limitations of the algorithm in situations where more precise boundaries are required for the definition of correct detections and, therefore, these results signal the need to direct efforts toward improving and refining the model, seeking to improve its detection capacity in more complex scenarios with greater overlap between objects. Similar results were found in the work of [47], which also found a lower performance for the mAP 0.95 metric, especially in the IoU threshold range between 0.5 and 0.95.

The characteristics and technical specifications of the model used in the research were also analyzed, and the results reveal that the yolov5m model requires a computational power of 47.9 GFLOPs. Meanwhile, the yolov5s model requires a computational power of 15.8 GFLOPs and the yolov5x model requires a higher computational power, reaching 203.9 GFLOPs. Similar results were obtained by [68,69], who also obtained a value of 15.8 GFLOPs in their experiments using yolov5s. On the other hand, the study [70] revealed a slightly higher value of 16.5 GFLOPs. It is important to note that [47] were able to significantly reduce the required computing power, from 15.8 to 4.7 GFLOPs, by using MobileNetV3-Large as a replacement for YOLOv5s’ CSPDarknet53 backbone network in their respective studies. These findings point to potential optimizations that can be applied to the model to improve its computational efficiency without compromising detection performance.

In addition, the average time required to run the model’s inference on each image was also observed, that is, the time for the model to process the input data and generate the object detection predictions. For YOLOv5m it was 1.5 ms, whereas for YOLOv5s and YOLOv5x it was 2.8 ms, allowing the model to analyze 7.70 images per second. 

For the application of YOLOv5 in smart livestock farming, especially for tasks such as the evaluation of animal behavior and health, one must consider the specific requirements related to network size, detection speed, and computational efficiency, due to the dynamic and real-time nature of the operations performed, as suggested by [8] for the implementation of YOLOv5 in automation systems and intelligent robots. 

The results from the analysis of variance (ANOVA) indicate that there is no substantial evidence indicating a significant difference in the mean performance between the YOLOv5s, YOLOv5m and YOLOv5x models for the behavioral classes, at the significance level of 5% for precision (*p*-value = 0.567), recall (*p*-value = 0.714), F-measure (*p*-value = 0.675) and accuracy (*p*-value = 0.682). 

However, when comparing the models and considering the production requirements of the swine industry, the YOLOv5s was selected because it had the highest mAP50-95 and the lowest number of parameters, making the model lighter.

**Figure 8:**
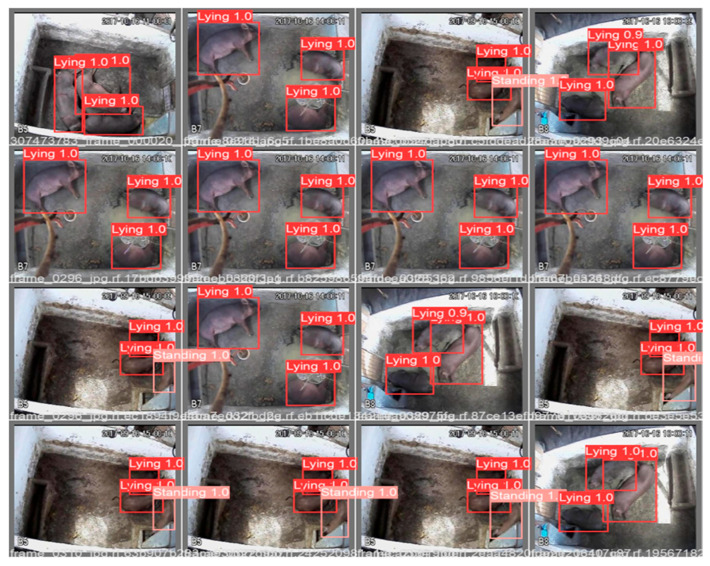
Result of YOLOv5 detections.

After training, the model detects each animal individually when it receives an image. Each detection result is marked with a bounding box, which has a label and a number indicating its probability of belonging to that label, as shown in Figure 8.

The observation of Figure 8 revealed that the probabilities attributed by the model to the lying or standing classes ranged from 0.9 to 1, indicating that the model was able to differentiate between these categories of behavior.

#### 3.3.2. Identification of Behavior Patterns in Pigs

**Figure 9:**
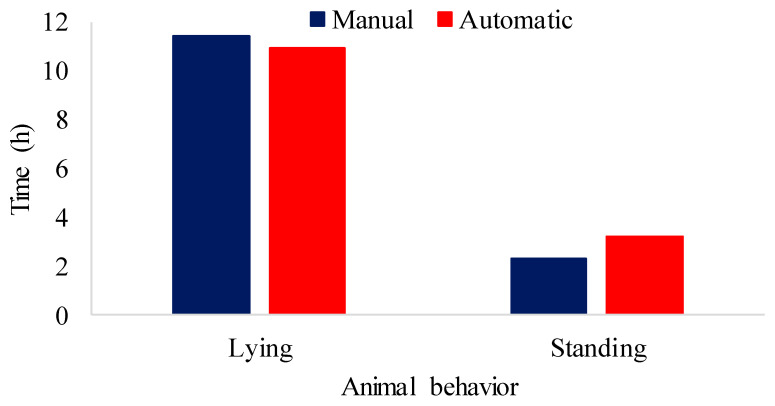
Manual and estimated monitoring in the bays with air conditioning.

**Figure 10:**
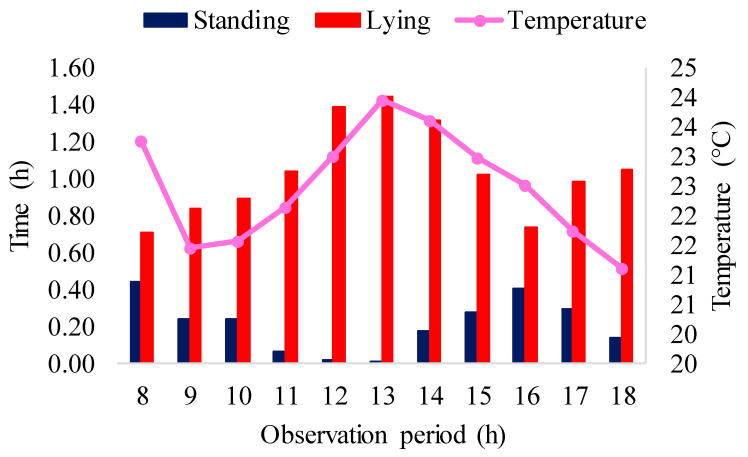
Behaviors in the air-conditioned environment.

**Figure 11:**
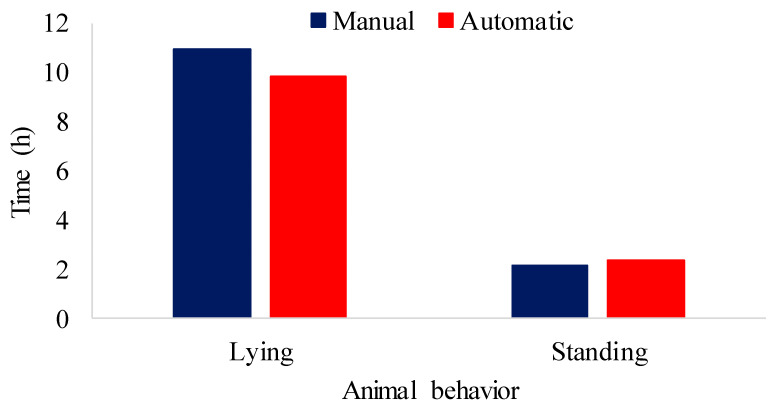
Manual and automatic monitoring in the non-air-conditioned bays.

**Figure 12:**
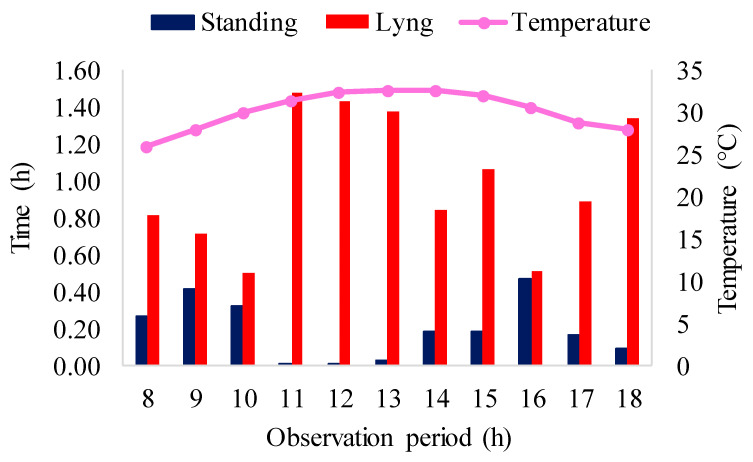
Behaviors analyzed in the non-air-conditioned environment.

To confirm the validity of the automatic detections, all video segments were independently annotated by two trained human observers who were blinded to the model’s outputs. The observers recorded the start and end times of standing, lying, eating, and drinking behaviors using a custom annotation interface. Inter-observer agreement was high (Cohen’s κ = 0.92). The human-annotated data served as the ground truth against which the YOLOv5 predictions were compared. The differences reported in Figure 9, Figure 10, Figure 11 and Figure 12 and Table 12 and Table 13 are therefore direct measures of the model’s accuracy relative to manual observation.

Figure 9 shows the results of manual monitoring and automatic monitoring of pigs submitted to air-conditioned pens during the experimental period for lying down and standing behaviors.

The biggest difference found between manual and automatic monitoring was for the standing class, including investigation and locomotion activities, as pigs stand while investigating or moving. The maximum difference found was 0.933 h. Manual monitoring revealed that the animals stood for 2.282 h, while automatic monitoring showed 3.215 h (20.03%). In the lying down class, manual monitoring showed that the animals were lying down for 11.384 h, while the model revealed 10.913 h (68.02%), a difference of 0.47 h.

These observed differences between manual and automatic measurements can be attributed to the images in the dataset. In some videos, the lighting compromised the model’s ability to discern between the stall floor and the animals that were lying or sleeping on it, especially due to the similarity in color, since the stall floor had nearly the same color as the animals. The floor soiled with animal excreta also contributed, since the model, by detecting dirty areas, misinterpreted these regions as animals, thus influencing the behavior estimates. In addition, a second unused feeder was installed in the facilities, whose color resembled that of the animals, resulting in incorrect detections, with the model mistakenly identifying the feeder as an animal. In the research of [71], that used YOLOv5 to detect the posture of pigs, the model missed detections and made erroneous detections due to lighting problems that hid the animals. 

Figure 10 shows the lying and standing behaviors in the stalls with air conditioners associated with the ambient temperature values.

The results of Figure 10 show that the animals in the air-conditioned environment spent most of the time, about 11.38 h (68.02%), lying down, especially at the time of highest temperature (12 and 1 pm). In addition, for 2.28 h (20.03%) of the time the animals developed standing activities, such as investigation and locomotion. This behavior suggests that pigs prefer to lie down most of the time in an air-conditioned environment, which may indicate a higher level of comfort and well-being in this type of environment. In the work of [71], pigs slept about 70% of the time analyzed, with a higher proportion of pigs lying down from 12 pm to 1 pm.

In the work of [72], pigs spent most of their time lying down (50%) when exposed to temperatures within the thermoneutral zone (18 to 21 °C). This shows that environmental conditions significantly impact behavior. Ref. [73] highlighted that pigs are highly sensitive to changes in ambient temperature and perform better when kept in a comfortable thermal environment, while ref. [74] observed that the animals explored the environment more when the air temperature was in the thermoneutral zone. 

Figure 11 represents the results of monitoring the behavior of animals in stalls without air conditioning. These observations come from both manual and automatic evaluations.

The results of Figure 11 reveal that the animals in the natural environment spent most of the time lying down, with a manual evaluation recording 10.96 h and a model evaluation showing 9.84 h (69.71%), resulting in a difference of 1.12 h. In addition, the animals performed activities while standing, such as investigation and locomotion, for 2.14 h, while the model evaluation indicated 2.36 h (16.78%), resulting in a difference of 0.22 h. Ref. [74] observed that the animals explored the environment more when the air temperature was in the thermoneutral zone. In the present study, the non-air-conditioned stalls were in thermal discomfort with temperature values higher than the thermoneutral zone (18 to 26 °C), so this activity was less observed among the animals housed in the stalls without air conditioning.

Regarding the difference in the lying class between the observed and the estimated, this disparity is attributed to the limitation of the model in differentiating the animals lying on the ground, similar to the air-conditioned stalls, due to the similarity in coloration. In situations where the animals overlapped, detections were lost, reflecting a limitation in the model’s capability. This limitation was also identified by [64] in its chicken detection work, where YOLOv5 missed detections due to overlapping and crowding of the animals. In the research of [71], when part of the pig’s body was missing or blocked by obstacles such as lighting, YOLOv5 lost detection of the pigs. In the study of [75], the authors encountered challenges related to lighting, YOLOv5 missed piglet detections and obtained false detections in interference scenarios, with intense light.

As for the standing class, the performance of the detections may have been affected by the presence of the unused feeder in the stall and the wet floor due to the excreta of the animals, which resulted in a darker shade on the floor (similar to the animals in the air-conditioned stalls). These elements may have contributed to false detections, as observed in the work of [23], which used YOLOv5 to detect and count pigs, where the model also mistook the feeder for an animal. This is because the model tends to emphasize the color of the target and not the shape. In the study conducted by [71], challenges were identified in the detection of lying postures in pigs. This problem arose due to the proximity of the pig’s head to the ground, resulting in the concealment of the front legs, which are essentially hidden in front of the breast. These unfavorable lighting conditions further hampered the model’s ability to accurately identify the animals when they were in the lying position. In the research of [64], false detections were also identified by YOLOv5 in the presence of equipment in the birds’ facilities. 

Figure 12 presents the visual representation of the lying and standing behavior observed in the stalls without air conditioners, in association with the air temperature, allowing a view of the behavioral responses to the temperatures.

Automatic monitoring reveals that the animals spent most of their observation time at rest. The lying activity was the one that totaled the longest time, about 9.84 h (69.71%) of the time analyzed, and the time that the animals spent the longest time lying down was from 11 to 13 h and at the end of the day, at 6 pm. This behavioral pattern is often observed in pigs that are exposed to hot environments as a strategy for heat loss through contact with the floor. Since pigs do not have the ability to sweat, they adopt specific strategies to cope with high temperatures, such as resting on cooler surfaces to regulate body temperature [60]. In addition, the research conducted by [76] revealed that these animals spent most of their time at rest, highlighting that in his study with lactating sows, the pigs spent about 44.30% of the day sleeping. In the research of [67], the researchers highlighted that pigs are prone to prolonged periods of sleep, corroborating this observation.

Standing behavior required 2.36 h (16.78%) and walking and investigating behaviors were included in this category. This behavior is in accordance with the study carried out by [77], which identified that, in the growing and finishing phases of pigs, exploratory behavior was the second most frequent, being surpassed only by the lying behavior. These behaviors are useful for measuring the health and welfare of farm animals, according to [78].

### 3.4. Eating and Drinking Behavior

The behavior of food and water intake was analyzed in the video segments recorded after feeding the pigs, performed twice a day (morning and afternoon). Water consumption was analyzed after feeding, considering that the animals tended to eat and then go to the drinker. The accuracy and recall metrics were used to calculate prediction errors and evaluate the performance of the proposal shown in Table 12. The ground truth for eating and drinking was established by the same two human observers, who visually inspected the video segments frame-by-frame to record each drinking or eating event. Only events where the pig’s snout was clearly in contact with the drinker or feeder were counted. The model’s detections (based on bounding-box intersection with the regions of interest) were then compared against this manual annotation.

As can be seen, for the prediction results based on 30-min time intervals, the model obtained accuracy and recall of 0.97 and 0.96, respectively, with mean values of 0.975 and 0.970. In the eating class, the model obtained accuracy and recall of 0.98. This indicates strong agreement between the observed and predicted values. The results of these studies are similar to those obtained by [79], which demonstrated good performance in recognizing the feeding behavior of pigs in the test set, achieving an accuracy of 0.984, a recall of 0.988 and an average accuracy of 0.959.

In the research conducted by [80], anaccuracy of 0.944 was observed in the recognition of feeding behavior in pigs housed in commercial pens, using the YOLOv5 model.

Ref. [81] used YOLOv5 to detect eating and drinking behaviors in goats and achieved average accuracies of 0.978 and 0.982, respectively. Ref. [49] used YOLOv5 to verify the feeding behavior of pigs and achieved a recognition accuracy of 0.994. Ref. [82] used a deep learning network to recognize the drinking and feeding behaviors of cows and achieved an average accuracy of 0.978. 

Table 13 presents data on the time used by animals for water intake and feed intake by the animals in the air-conditioned stalls and in the pens without air conditioners.

For drinking behavior, the manual method recorded an average of 0.067 h in the air-conditioned environment, while the automatic method indicated 0.063 h (0.39%). In the natural environment, manual observation recorded 0.103 h, while the automatic method recorded 0.1636 h (1.15%). For eating behavior, the manual method recorded an average of 1.790 h in the air-conditioned environment, while the automatic method recorded 1.8518 h (11.54%). In the natural environment, manual observation recorded 1.6674 h, while the automatic method recorded 1.7419 h (12.33%). This may have occurred due to the lack of discontinuity between frames, since the animal may no longer be drinking, however, its bounding box may still occupy the entire region of the drinker. 

**Figure 13:**
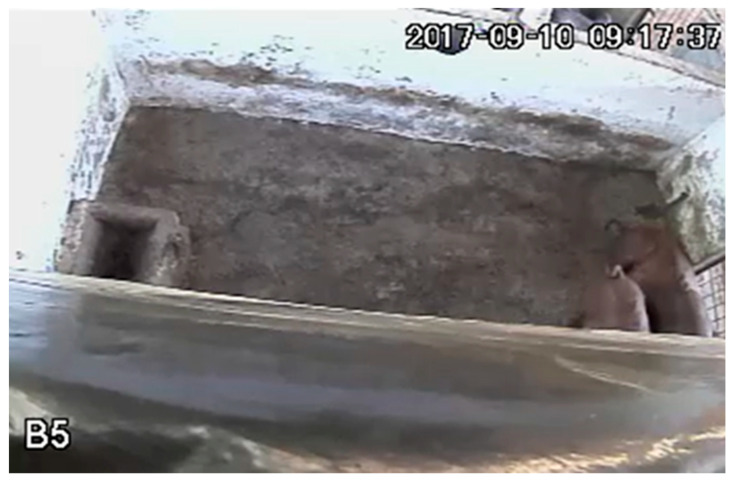
Illustration of tarpaulin in non-air-conditioned stalls.

The results indicate that YOLOv5 is able to identify the behavior of animals, although there are slight variations in measurements between methods. The smallest difference (0.0043) occurred for the drinking class in the air-conditioned environment, where manual observation detected 0.0678h and the model detected 0.0635h. The biggest difference occurred for the eating class (0.0745) in the natural environment. This difference may be associated with the presence of a tarpaulin that was placed to avoid interference from the air-conditioned environment and may have confused the model, causing difficulties for the algorithm detecting more animals at the feeder (Figure 13).

In the videos with better lighting in the animal facilities, the pigs become more prominent, so they were better identified by the model, which was therefore able to discriminate them more accurately. The high accuracy of the model under such lighting conditions makes it easy to monitor the animals during the day.

Still observing Table 13, it can be seen that the animals in the air-conditioned environment required around 0.0678 h of water intake, while the animals in the natural environment drank around 0.1031 h. The results obtained indicate that the animals in the air-conditioned environment drank less water, with a reduction of approximately 34.56% compared to those in the natural environment (0.0678 h in the air-conditioned environment compared to 0.103h in the natural environment). This decrease can be interpreted as an adaptive response to the colder environment, suggesting that the need for hydration may be lower under these conditions. This is because the animals in the stalls without air conditioners were under temperatures higher than the thermoneutral zone and, according to [83], when animals are subjected to temperatures higher than their thermal comfort zone, water intake increases and food consumption decreases. 

The animals housed in these stalls had a lower water consumption (0.11 h) (0.68%) compared to the animals in the stalls without air conditioners. Ref. [52] also identified this trend, noting that in environments with a temperature higher than 26 °C, pigs demonstrated an increase in water consumption. These findings corroborate the results identified by [74] who also found that pigs in hot environments ingest more water. This behavioral pattern is in line with the observations of this study, where the animals in the air-conditioned environment showed a greater propensity to remain standing and make more visits to the drinker. 

Regarding eating, the animals in the air-conditioned environment had an average of 7.42% more time dedicated to this activity compared to those in the natural environment (1.7909 h in the air-conditioned environment compared to 1.6674 h in the natural environment). This difference suggests a possible response to different environments, indicating that climatic factors can influence the feeding patterns of animals. 

These findings corroborate the study by [84] who also investigated the behavior of pigs in both air-conditioned and non-air-conditioned environments and observed the same behavioral pattern. In the research of [3], actating sows ate for 1.06% of the time analyzed. Ref. [85] state that this observation is associated with the microclimate of the environment, since pigs exposed to high temperatures show a reduction in feed intake.

These results were similar to those found by [86], who analyzed the behavior of pigs in an air-conditioned environment and identified that the animals consumed more feed in this condition. Ref. [87] described that in high temperature environmental conditions, pigs tend to reduce their feed intake and reduce their visits to the feeder. A similar behavioral pattern was observed by [88], who observed that animals at the right temperature eat more and drink less water. These behaviors, when compared with the data on the physiological variables, are correlated, since the animals in the air-conditioned stalls presented the lowest values of the physiological variables.

Ref. [83] observed that the consumption of feed in stalls whose temperatures were within the range of thermal comfort for pigs in the growing and finishing phases was higher compared to stalls subjected to higher temperatures. Thus, it was observed that the air-conditioned environment contributed to the reduction of heat stress and, consequently, to the increase in feed consumption. 

On the other hand, drinking was the activity that required the shortest time (0.0635 h) and was the least observed compared to the other activities. Drinking behavior in pigs is of short duration and, according to [4], is occasional. This observation is also consistent with the findings of [3], who also monitored the behavior of lactating sows and observed that the animals had short periods of water intake (1.63%).

The animals in the air-conditioned stalls ate more, drank less water, stayed longer on their feet, and slept more than the animals in the stalls without air conditioners. These behaviors, when compared with the data of the physiological variables, are correlated, since the animals in the air-conditioned stalls presented the lowest values of the physiological variables. 

The behavioral metrics derived from the YOLOv5 model can be directly interpreted in the context of thermal stress. Under heat stress conditions (non-air-conditioned pens: average BGHI 79.8, respiratory rate > 60 mov·min^−1^), the model detected a 7.4% reduction in feeding time and a 34.6% increase in drinking time compared with the thermoneutral environment. These changes are consistent with known physiological responses: pigs reduce feed intake to decrease metabolic heat production and increase water consumption to support evaporative cooling. Moreover, the model’s detection of lying behavior (69.7% of the time in heat-stressed pens vs. 68.0% in thermoneutral pens) aligns with the strategy of maximizing body contact with cooler floor surfaces. By providing continuous, automated quantification of these behaviors, the YOLOv5 model offers a practical tool for early detection of heat stress before rectal temperature rises above critical thresholds. This integration demonstrates that computer vision is not merely a replacement for manual observation but can generate biologically meaningful indicators of animal welfare in real time.

**Figure 14:**
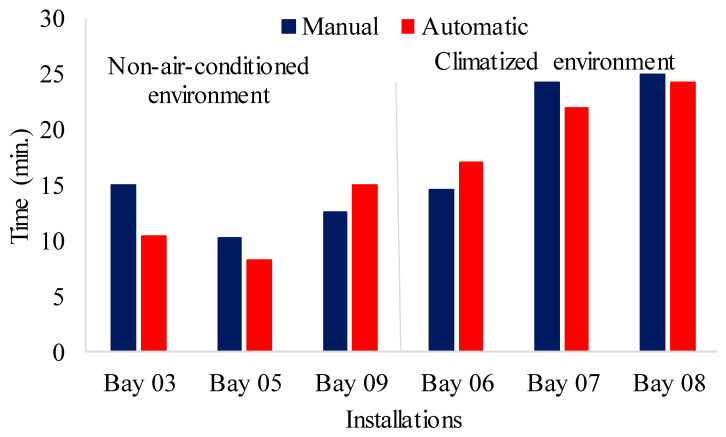
Visual and automatic monitoring of food intake in stalls.

To verify the generalizability of the algorithm in recognizing the eating and drinking behavior of pigs, the 30-min videos were divided into 5-min segments of each of the experimental facilities, as shown in Figure 14.

Videos of each of the stalls after the feed was supplied were selected for verification purposes to avoid the possibility of the animal investigating the feeder without having food. The results in Figure 14 show that the proposed method effectively detects the eating activities of the animals in each frame of the video. The proposal can monitor pigs to continuously verify the occurrence of feeding behavior. These results suggest that, under the conditions tested (two distinct housing environments (air-conditioned and naturally ventilated), with a fixed camera setup and controlled lighting), the model shows promising potential for application in conventional breeding environments. Further validation on larger datasets from different farms, seasons, and camera positions would be needed to fully assess its generalizability.

Although human observation is itself subject to fatigue and minor errors, the high inter-observer agreement and the use of blinded annotation minimize bias. The relatively small discrepancies between manual and automatic measurements are within acceptable ranges for behavioral studies and are comparable to those reported in similar YOLOv5-based livestock monitoring studies [66,73]. Thus, the manual validation approach provides a robust ground truth for evaluating the algorithm’s performance.

The results show that most of the animals in the stalls without air conditioners spend less time in the feeder. When associating these behaviors with environmental variables, it is observed that these stalls had higher temperature and lower humidity. The monitoring data using the proposed automated system clearly demonstrated that drinking behavior reflects the effects of heat stress on pigs. According to [33], animals subjected to higher temperatures reduce feed consumption. The thermoneutral temperature in the finishing phase is 18 °C, and the temperature causing heat stress is higher than 27 °C. The average temperatures in these stalls were above 27 °C.

Pig monitoring is an important part of smart animal husbandry. Monitoring animals automatically can help farmers improve production and avoid wasting time. However, the lighting system, installed equipment, and the color of the floor of the facility can pose many challenges for swine detection.

## 4. Conclusions

Lying and standing behaviours are indicators of the well-being and health of pigs in collective environments. The YOLOv5 model, trained to identify these behaviors, demonstrated high efficiency, achieving an average accuracy rate of 97.3% and an average recall of 96.1%. In addition to accurately detecting standing and lying pigs, the results showed that the model was able to identify behavioral differences associated with the climatic conditions of the housing. Specifically, pigs housed in stalls without air conditioners spent less time standing, while those in air-conditioned pens spent more time standing. These findings indicate that the YOLOv5 model is a reliable tool to monitor pigs in real time, promoting more appropriate, healthy and efficient breeding practices.

The algorithm exhibited average accuracy rates of 97.5% and an average recall of 97.0% in recognizing the feeding behavior and water consumption of pigs. The results showed that the model was able to identify the behaviors of the animals associated with climate, since the animals in the stalls without air conditioning spent less time at the feeder, while the animals in the air-conditioned stalls spent more time there. The proposal can be used in surveillance videos or images and minimizes the need for manual intervention, offering an efficient means of monitoring the behavior of pigs in agricultural environments and contributing to the productivity of pig farming operations, ensuring more suitable and healthy conditions for pigs, as well as promoting more sustainable and efficient farming practices.

## Figures and Tables

**Table 1:** Pig behavior.

Behaviors	Labels	Description
Drinking	Drinking	Pig ingesting water from the drinker
Eating	Eating	Pig ingesting food from the feeder
In bed	Lying	Pig lying or sleeping on the floor
Standing	Standing	Pig moving or investigating

**Table 2:** Optimal and critical temperatures and relative humidity for pigs.

Pigs	Optimal Temperatures (°C)		Recommended Temperatures (°C)		Relative Humidity (%)	
	Maximum	Minimal	Maximum	Minimum	Good	Critical
Growth	21	18	30	8	70	<40 e >90
Termination	18	15	27	5		

**Table 3:** Respiratory rate and rectal temperature.

Pigs	Respiratory Rate (Mov·min^−1^)		Scrap Temperature (°C)	
Category	Normal	Heat stress	Normal	Heat stress
Adults	≤60	>60	≤39.3	>39.3

**Table 4:** Black Globe Humidity Index (BGHI) classification for Pigs.

Category	Interval
Stress	BGHI ≤ 67
Comfort	68 < BGHI ≤ 74
Alert	75 < BGHI ≤ 76
Emergency	BGHI > 76

**Table 5:** Model size and training duration.

Model	Model Size (MB)	Time without GPU (h)	Time with GPU (h)
Yolov5s	27	3.50	1.52
Yolov5m	45	5.40	1.82
Yolov5x	173	10.00	2.70

**Table 6:** Air temperature and relative humidity in the facilities.

Treatments	Air-Conditioned		Non-Air-Conditioned	
Parameters	T Air (°C) ^1^	RH (%) ^2^	T Air (°C) ^1^	RH (%) ^2^
Average	22.59	81.99	30.43	54.62
Minimum	21.02	70.30	24.56	46.62
Maxim	24.87	85.73	33.52	74.63

^1^ Air temperature and ^2^ Relative humidity.

**Table 7:** Results of physiological variables.

Timetable (Hours)	Bays with Air Conditioners		Bays Without Air Conditioners	
	RT ^1^ (°C)	RR ^2^ (Mov·min^−1^)	RT (°C)	RR (Mov·min^−1^)
08:00	38.33	44.67	38.75	48.33
12:00	38.40	59.67	38.50	73.50
16:00	37.90	55.67	38.57	70.33

^1^ Rectal temperature and ^2^ Respiratory Rate.

**Table 8:** BGHI of the stalls.

BGHI	Stall with Cooling System	Stall Without Cooling System
Mean	71.45	79.82
Minimum	69.12	73.63
Maximum	74.80	83.17

**Table 9:** Evaluation metrics in the yolov5m test suite.

Class	P	R	mAP50	mAP50-95
In bed	0.982	0.980	0.992	0.948
Standing	0.948	0.942	0.977	0.784
Average	0.965	0.961	0.984	0.866

**Table 10:** Evaluation metrics on the YOLOv5s test set.

Class	P	R	mAP50	mAP50-95
In bed	0.984	0.981	0.985	0.975
Standing	0.962	0.941	0.985	0.952
Average	0.973	0.961	0.985	0.963

**Table 11:** Evaluation metrics in the yolov5x test suite.

	P	R	mAP50	mAP50-95
In bed	0.987	0.984	0.993	0.960
Standing	0.962	0.941	0.969	0.744
Average	0.974	0.962	0.981	0.852

**Table 12:** Performance metrics.

Metric	Drinking	Eating	Average
P	0.97	0.98	0.975
Recall	0.96	0.98	0.970

**Table 13:** Water and food intake of animals in the studied environment.

Activities	Heated		Non-Air-Conditioned	
	Manual	Automatic	Manual	Automatic
Drinking	0.0678 h	0.0635 h	0.1031 h	0.1636 h
Eating	1.7909 h	1.8518 h	1.6674 h	1.7419 h

## Data Availability

The datasets used in this study comprise a personal archive that is part of ongoing and future research. Data availability may be considered upon request from readers, subject to evaluation.
